# Peptidylarginine Deiminase 3 (PAD3) Is Upregulated by Prolactin Stimulation of CID-9 Cells and Expressed in the Lactating Mouse Mammary Gland

**DOI:** 10.1371/journal.pone.0147503

**Published:** 2016-01-22

**Authors:** Guangyuan Li, Isaac N. Hayward, Brittany R. Jenkins, Heather M. Rothfuss, Coleman H. Young, Marja T. Nevalainen, Aaron Muth, Paul R. Thompson, Amy M. Navratil, Brian D. Cherrington

**Affiliations:** 1 University of Wyoming, Department of Zoology and Physiology, Laramie, WY, United States of America; 2 Medical College of Wisconsin, Department of Pathology, Milwaukee, WI, United States of America; 3 University of Massachusetts Medical School, Department of Biochemistry and Molecular Pharmacology, Worcester, MA, United States of America; CNRS-University of Toulouse, FRANCE

## Abstract

Peptidylarginine deiminases (PADs) post-translationally convert arginine into neutral citrulline residues. Our past work shows that PADs are expressed in the canine and murine mammary glands; however, the mechanisms regulating PAD expression and the function of citrullination in the normal mammary gland are unclear. Therefore, the first objective herein was to investigate regulation of PAD expression in mammary epithelial cells. We first examined PAD levels in CID-9 cells, which were derived from the mammary gland of mid-pregnant mice. PAD3 expression is significantly higher than all other PAD isoforms and mediates protein citrullination in CID-9 cells. We next hypothesized that prolactin regulates PAD3 expression. To test this, CID-9 cells were stimulated with 5 μg/mL of prolactin for 48 hours which significantly increases PAD3 mRNA and protein expression. Use of a JAK2 inhibitor and a dominant negative (DN)-STAT5 adenovirus indicate that prolactin stimulation of PAD3 expression is mediated by the JAK2/STAT5 signaling pathway in CID-9 cells. In addition, the human PAD3 gene promoter is prolactin responsive in CID-9 cells. Our second objective was to investigate the expression and activity of PAD3 in the lactating mouse mammary gland. PAD3 expression in the mammary gland is highest on lactation day 9 and coincident with citrullinated proteins such as histones. Use of the PAD3 specific inhibitor, Cl4-amidine, indicates that PAD3, in part, can citrullinate proteins in L9 mammary glands. Collectively, our results show that upregulation of PAD3 is mediated by prolactin induction of the JAK2/STAT5 signaling pathway, and that PAD3 appears to citrullinate proteins during lactation.

## Introduction

Peptidylarginine deiminases (PADs or PADIs) are a family of calcium dependent enzymes that convert positively charged arginine amino acids to the neutral residue citrulline. This post-translational modification alters protein charge resulting in changes in protein structure and molecular interactions. The PAD enzyme family has a highly conserved genomic organization on human chromosome 1 and on an orthologous region of mouse chromosome 4 [1]. There are 5 PAD family members: PADs 1, 2, 3, 4 and 6. Although PAD enzymes sometimes display overlapping tissue expression patterns, each family member has distinct substrate specificities. A notable exception to this is PAD6, which does not appear to have catalytic activity [2]. Accumulating evidence indicates that PAD enzymes function in human diseases such as lupus, multiple sclerosis, ulcerative colitis, rheumatoid arthritis and cancer [3–8]. Despite this increased attention, the mechanisms that regulate PAD expression and their normal physiological functions remain unclear in many tissues.

Our previous findings in the canine mammary gland link PAD expression with pregnancy and lactation [9]. During canine pseudopregnancy, elevated serum prolactin can induce active lactation by the mammary gland. Interestingly, during this time PAD2 expression levels are the highest in the canine mammary gland. Further evidence linking PADs with pregnancy and lactation is a report showing that PAD activity substantially and steadily rises in rat anterior pituitary gland lactotrope cells from day 7 of pregnancy through day 14 [10]. Based on these findings, it is highly probable that pregnancy and lactation associated hormones may regulate PAD expression in lactotrope and mammary secretory cells, and a potential candidate for this regulation is prolactin. To initiate lactation, high levels of serum prolactin bind to prolactin receptors located on the mammary secretory cell membrane. The prolactin receptor, a member of the type I cytokine receptor family, activates the Janus Kinase 2 (JAK2)/Signal Transducer and Activator of Transcription 5 (STAT5) signaling pathway. Once activated, phosphorylated STAT5a and b dimerize, translocate to the nucleus, and target interferon-γ-activated sequence (GAS) motifs on lactation related gene promoters dramatically increasing breast milk production by mammary secretory cells. For example, prolactin is required for stimulating robust transcription of a cohort of genes that encode proteins necessary for milk synthesis and secretion such as butyrophilin and α-lactalbumin [11, 12].

In breast cancer cell lines, PADs 2 and 4 participate in the epigenetic control of gene expression, and both isoforms are expressed in human breast tumors [7, 13–15]. However, regulation of PAD expression in the normal mammary gland and related cell lines is not well understood. In fact, all that is currently known is that expression of PADs 2 and 4 changes across the estrous cycle in mouse mammary tissue [16]. In an attempt to address this deficit in our knowledge, we first examined PAD expression levels in CID-9 cells which were isolated from the mammary epithelia of a mid-pregnant mouse [17]. Surprisingly, the expression of PAD3 mRNA was significantly higher than other isoforms, yet expression of PAD3 in the mammary gland has never been described. PAD3 is best characterized in keratinocytes where it citrullinates cytokeratin K1, K10, and filaggrin to promote epidermal homeostasis and barrier formation [18]. Citrullinated filaggrin interacts with cytokeratins to form cross-linked structures during terminal differentiation of keratinocytes during cornification [19]. In the inner root sheath and cuticle of hair follicles, PAD3 citrullinates trichohyalin strengthening the inner root sheath allowing for directional hair growth [20, 21]. Recent evidence indicates that PAD3 also functions in systems other than skin physiology. In human neuronal stem cells, Pong *et al*. showed that PAD3 activity is required for apoptosis inducing factor (AIF) mediated apoptosis and calcium dependent cytoskeletal organization [22]. Given that PAD3 is expressed in CID-9 cells, we investigated how it is regulated and if it is expressed in lactating mouse mammary glands.

Herein, we report that prolactin treatment of CID-9 cells induces PAD3 expression via the JAK2/STAT5 signaling pathway. Prolactin responsiveness localize to a 182 base pair region in the proximal human *PAD3* gene promoter. Next, we investigated the physiological relevance of this observation and found that PAD3 expression in the mouse mammary gland initiates at pregnancy day 18 (P18) and is highest on lactation day 9 (L9). Consistent with high PAD3 levels, citrullinated proteins are present in lactating mouse mammary glands. Inhibition of PAD3 by Cl4-amidine in L9 mammary gland lysates indicates that PAD3 catalyzed citrullination occurs during lactation. Overall, our work defines a novel signaling pathway regulating PAD expression and supports a functional role for PAD3 in the lactating mammary gland.

## Material and Methods

### Cell culture

Dr. Mina Bissell generously provided the CID-9 cell line which was maintained in DMEM/F12 (HyClone) supplemented 1% penicillin-streptomycin (Sigma-Aldrich, St. Louis, MO), 5 μg/mL insulin (Sigma-Aldrich), and 2% fetal bovine serum (FBS) (HyClone) [17]. The COMMA-1D cell line was generously provided by Dr. Daniel Medina and grown in DMEM/F12 (HyClone Laboratories, Inc., South Logan, UT) supplemented with 10 μM HEPES buffer (Sigma-Aldrich), 1% penicillin-streptomycin (Sigma-Aldrich), 10 μg/mL insulin (Sigma-Aldrich), 5 ng/mL EGF (Sigma-Aldrich), and 2% FBS (HyClone) [23]. Cells were maintained in a humidified atmosphere of 5% CO_2_ at 37°C. For prolactin treatment experiments, CID-9 cells were grown overnight in phenol red free DMEM (HyClone) with 2% charcoal stripped FBS (Corning, Mediatech, Inc., Manassas, VA). The following morning, cells were treated with vehicle or 5 μg/mL of prolactin (Sigma-Aldrich) for 24 or 48 hours. For inhibitor studies, cells were pre-treated with 3 μM JAK2 inhibitor, SD-1029 (EMD Chemicals, Inc., San Diego, CA) for 4 hours prior to prolactin treatment described above. Dr. Marja Nevalainen generously provided the dominant negative STAT5 adenovirus (Ad-DN-STAT5) [24]. CID-9 cells were infected with adenovirus (MOI = 10) expressing Ad-DN-STAT5 or GFP (Ad-GFP) as a control. Following an overnight infection, cells were treated with vehicle or prolactin for 24 or 48 hours as described above. Dr. Paul Thompson generously provided the PAD3 inhibitor, Cl4-amidine[25]. CID-9 cell were treated with vehicle or 50 μM Cl4-amidine overnight.

### Mouse mammary tissue

FVB mice were maintained on a 12 hour light/dark cycle with *ad libitum* access to food and water. Following mating with an FVB male, the presence of a copulatory plug was considered day 1 of pregnancy. Mouse mammary tissue was collected from 6 female mice on pregnancy days 12 and 18 and lactation days 2 and 9. Euthanasia was performed by CO_2_ asphyxiation and tissue harvested in accordance with the guidelines outlined in the Report of the AVMA on Euthanasia. All work in this study was approved by the University of Wyoming Institutional Animal Care and Use Committee (protocol #20140228BC00067-01).

### PAD3 vector construction

The 5’ flanking region of the *PAD3* gene was amplified by PCR using human genomic DNA as a template and PAD3 primers containing engineered XhoI and HindIII restriction enzymes sites as previously described [26]. Resulting promoter fragments (-276/+41 and -94/+41) were gel purified and cloned into pGEM-T Easy (Promega, Madison, WI). Fragments were released by XhoI/HindIII digestion and subcloned into pGL3-Basic cut with the same enzymes. The hPAD3–276/+41 and -94/+41-luciferase plasmids were verified by sequencing. The PAD3 cDNA was purchased from Open Biosystems (Hunstville, AL). The cDNA was removed from pCR-BluntII-TOPO by digestion with HindIII and XbaI. The resulting fragment was then ligated into pCDNA3.1+ previously digested with HindIII and XbaI and clones sequence verified.

### Immunohistochemistry (IHC) and immunofluorescence (IF)

IHC and IF experiments were carried out as previously described [9]. Slides were deparaffinized in 3 X 5 minute washes in xylene followed by sequential 5 minute rehydrations in 100, 95, and 75% EtOH. Endogenous peroxidases activity was blocked by incubating slides in 0.5% hydrogen peroxide in cold methanol for 10 minutes. Next, antigen retrieval was conducted by submerging the slides in 0.01 M sodium citrate and boiled 2X for 12 minutes. After cooling, slides were washed in 1X PBS and then blocked in 10% normal goat serum and 1X casein (Vector Labs, Burlingame, CA) diluted in 1X PBS for 20 minutes at room temperature in a humidified chamber. After removing excess blocking solution, slides were incubated with an anti-PAD3 (Antibodies-online Inc., ABIN347067, Atlanta, GA) or anti-citrulline (Abcam, ab6464, Cambridge, MA) diluted 1:100 in 1X PBS for 2 hours at 37°C. For negative controls, slides were incubated with an equal mass of non-specific rabbit IgG or without primary antibody but with secondary and tertiary reagents. After washing three times in PBS, slides were incubated for 20 minutes at room temperature with a biotinylated secondary antibody diluted 1:200 in 1X PBS. Following three washes in PBS, IHC slides were incubated in DAB chromagen (Vector Labs) solutions according to the manufacturer’s protocol, washed and then counterstained with hematoxylin and coverslip mounted. For IF, CID-9 cells were grown in MatTek 35 mm glass bottom dishes (MatTek, Ashland, MA). Following sequential steps with primary and biotinylated secondary antibodies, cells were incubated with streptavidin conjugated-488 (Vector Labs). After washing three times in 1X PBS, cells were stained with DAPI. For each experiment, duplicate dishes were incubated with an equal mass of non-specific rabbit IgG as a negative control.

### Western blotting

Cells were lysed with RIPA buffer containing 50 mM Tris, 150 mM NaCl, 0.1% SDS, 0.5% deoxycholate, 1% tritionX-100, 1 mM PMSF and 1X protease inhibitor (Thermo Scientific, Waltham, MA). Protein concentration in lysates was measured by BCA Assay prior to gel loading to ensure equal protein loading. 6X sample buffer consisting of 0.5 M Tris-HCl (pH 6.8), 60% glycerol, 30 mM DTT, 6% SDS was added into lysates to yield a final concentration of 1X sample buffer and lysates were then boiled at 95°C for 5 min. The lysates were subjected to SDS-PAGE using a 10% gel (acrylamide:bis-acrylamide ratio of 29:1) and subsequently transferred to Immobilin PVDF membranes (EMD Millipore, Billerica, MA). Membranes were blocked in 1X casein (Vector Labs) diluted in Tris buffered saline containing 0.1% Tween-20 (TBS-T) overnight at 4°C. Primary antibodies were incubated overnight at 4°C: Anti-PAD3 1:200 (Antibodies-online), anti-phospho-JAK2 (Cell Signaling Technology, #3774, Danvers, MA), anti-JAK2 (Cell Signaling Technology, #3230) anti-GFP (Cell Signaling Technology, #2956), anti-phospho-STAT5 (Cell Signaling Technology, #9359), anti-STAT5 (Cell Signaling Technology #9358), anti-citrullinated histone H3 arginine 2, 8, 17 (Abcam, ab5103) were all diluted 1:1000 and incubated overnight at 4°C. The following morning, membranes were washed in TBS-T, followed by a 2 hour incubation at RT with 1:15,000 anti-rabbit HRP secondary antibody (Jackson ImmunoResearch Labs, West Grove, PA). All blots were washed for 50 min (5×10 min) with TBS-T after secondary antibody incubation and then visualized using SuperSignal West Pico and Femto chemiluminescence substrate (Thermo Scientific). Anti-Modified Citrulline (AMC) western blots were performed according to the manufacture’s protocol (EMD Millipore). To confirm equal protein loading, all membranes were stripped and re-probed with anti-β-actin (Abcam, ab8227) or anti-β-tubulin (Sigma, T4023).

### qPCR

RNA was purified from CID-9 cells according to the Omega Bio-Tek Total RNA Kit protocol (Omega Bio-Tek, Inc., Norcross, GA). 1 μg of resulting RNA was reverse transcribed using iScript Reverse Transcription Supermix for RT-qPCR (BIO-RAD, Hercules, CA). Complementary DNA was subject to real time PCR analysis with SYBR Green assays (BIO-RAD) using intron spanning primers specific for PAD1, PAD2, PAD3, PAD4 or GAPDH and EEF2 as the reference gene control. The intron spanning primers used are listed in Table 1. Data was analyzed using the delta/delta Ct method in which Ct values of all target genes are adjusted to corresponding Ct value of reference gene (GAPDH or EEF2). All values are expressed as the mean ± SEM. Means were separated using ANOVA Tukey’s and * indicates significantly different means P<0.05.

**Table 1:** QPCR Primers.

	Primer sequence
PAD1	F: 5’ GGCAGAATCCTCATTGGTG 3’
PAD1	R: 5’ AGGAAGTCACGCACCACTCT 3’
PAD2	F: 5’ CCTCGAGATGGAAACCTGAA 3’
PAD2	R: 5’ GGGTCACATAGCCGAAATCT 3’
PAD3	F: 5’ CCCCTAGCAATGACCTCAAC 3’
PAD3	R: 5’ ATAGGCCAGGGGCAAATG 3’
PAD4	F: 5’ TGACCAATGGATGCAGGAC 3’
PAD4	R: 5’ CTCTGTCCCTCGGGGAGT 3’
GAPDH	F: 5’ GGGTTCCTATAAATACGGACTGC 3’
GAPDH	R: 5’ CCATTTTGTCTACGGGACGA 3’
EEF2	F: 5’ GTTGACGTCAGCGGTCTCTT 3’
EEF2	R: 5’ GCACGGATCTGATCTACTGTGA 3’

### Transient transfections and luciferase assays

DNA plasmids were purified by using E.Z.N.A plasmid DNA Midi Kit (Omega Bio-Tek) and transiently transfected into CID-9 cells using Mirus TransIT-2020 Reagent (Mirus Bio LLC, Madison, WI) according to the manufacturer’s protocol. Equal numbers of CID-9 cells were seeded on day one. On day two, cells were transfected with 0.4 μg of hPAD3-Luc -276/+41, hPAD3-Luc -94/+41 plasmids or empty pGL3 luciferase vectors as a control, and co-transfected with 0.1 μg of CMV β-galactosidase as a control for transfection efficiency. Following overnight transfection, cells were treated with 5 μg/mL of prolactin (Sigma-Aldrich) for 24 hours at which point they were lysed with reporter lysis buffer (Promega, Madison, WI). Luciferase and β-galactosidase activity was measured following the manufacturer’s protocol (Promega). Values were normalized for transfection efficiency by dividing the luciferase activity by β-galactosidase activity. All values are expressed as the mean ± SEM. Means were separated using Student’s T-Test and * indicates significantly different means P<0.05.

### PAD activity assay

Mouse mammary glands were lysed in modified RIPA buffer (50 mM Tris-Cl pH 8, 150 mM NaCl, 1%NP-40 alternative, 0.25% Na-deoxycholate), sonicated, and then cleared by centrifugation. The Color Development Reagent (COLDER) assay was carried out as previously described [25]. Briefly, 10 μg of total mammary gland protein was pre-incubated with vehicle or 100 μM Cl4-amidine for 15 min at 37°C. Cl4-amidine was generously provided by Dr. Paul Thompson [25]. The citrullination reaction was initiated by addition of 10 mM of benzoyl-L-arginine amide (BAA) (MP biochemicals, Santa Ana, CA). A parallel set of samples were incubated without BAA as a background control. All samples and controls (vehicle+BAA, vehicle-BAA, Cl4-amidine+BAA, and Cl4-amidine-BAA) were incubated overnight at 37°C, and the following morning citrulline levels were measured.

### Statistical analysis

All experiments were independently repeated at least three times and resulting values are expressed as the mean ± SEM. Means were separated using SNK and Tukey’s ANOVA or Student’s T-Test and letters or * indicates significantly different means P<0.05.

## Results

### PAD3 is the most highly expressed PAD isoform in mouse mammary epithelial CID-9 cells

**Fig 1:**
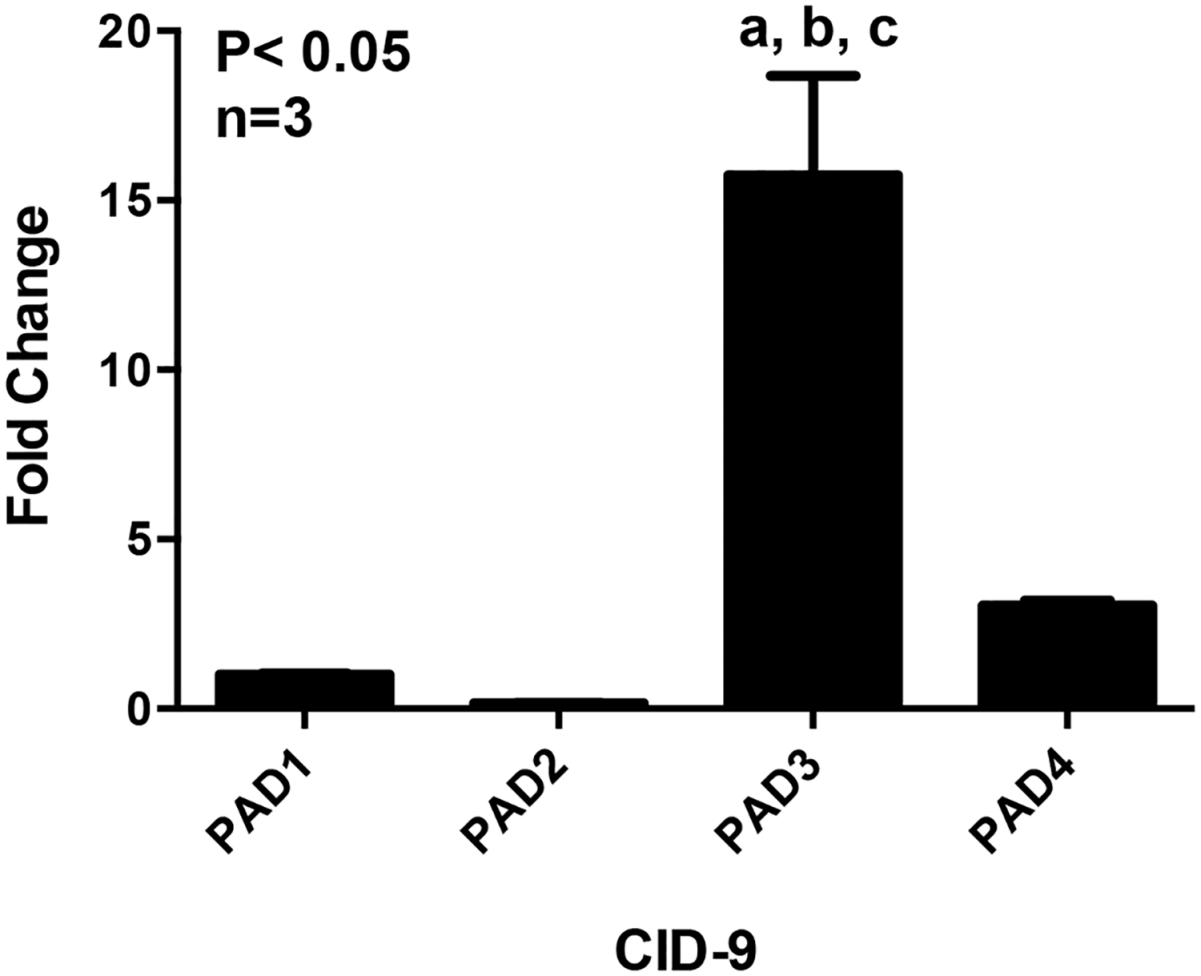
PAD3 is the most highly expressed PAD isoform in mouse mammary epithelial CID-9 cells. PAD3 mRNA levels are significantly higher in CID-9 cells compared to all other PAD isoforms. Total RNA was extracted from CID-9 cells, reverse transcribed, and resulting cDNA examined by qPCR with intron spanning primers specific for PAD1, PAD2, PAD3, PAD4 or GAPDH as the reference gene control. All data values were normalized to PAD1 to yield fold change, and data are expressed as means ± SEM. Means were separated using Tukey’s test ANOVA and letters indicate significant differences (P< 0.05).

In order to investigate PADs in normal mammary gland physiology, we first examined expression of PADs 1–4 in our model CID-9 cell line derived from the mammary epithelia of a mid-pregnant mouse. Surprisingly, PAD3 mRNA expression is significantly higher than any other PAD isoforms (P<0.05) and approximately 15 fold higher than PAD1 to which data is normalized (Fig 1). PADs 1–4 mRNA levels were also examined in another mouse mammary epithelial cell line termed COMMA-1D, which also shows that PAD3 mRNA levels are significantly higher than other isoforms (S1 Fig). This is the first study showing PAD3 expression in a mammary epithelial cell line.

#### Inhibition of PAD3 decreases the level of citrullinated proteins in CID-9 cells

**Fig 2:**
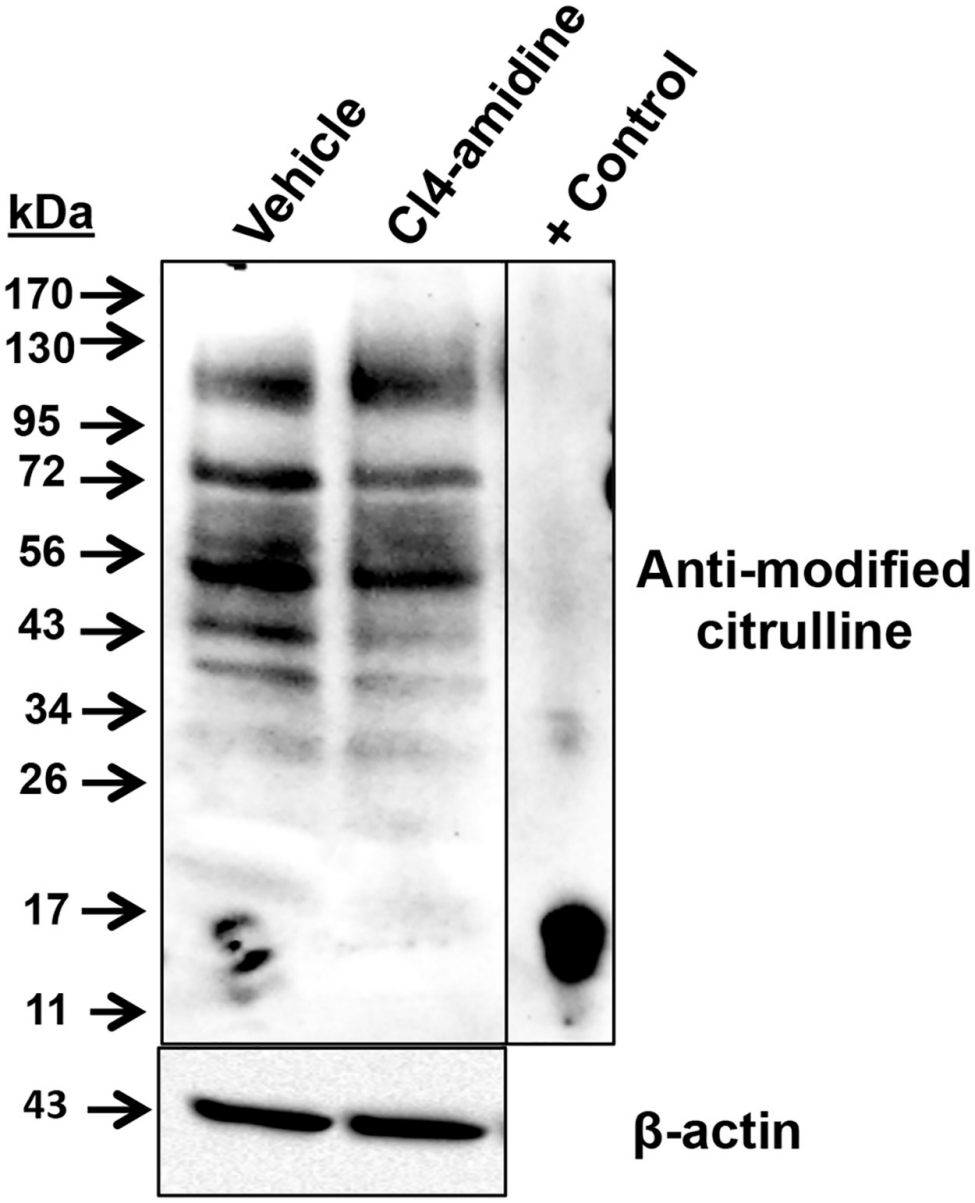
Inhibition of PAD3 decreases the level of citrullinated proteins in CID-9 cells. CID-9 cells were treated with vehicle or 50 μM Cl4-amidine overnight. The following morning cells were lysed and equal concentrations of protein lysates examined by western blot following the AMC protocol. The positive control is *in vitro* citrullinated bulk histones.

Given that PAD3 is highly expressed in CID-9 cells, we next investigated if it is catalytically active and citrullinates proteins. To determine this, CID-9 cells were treated overnight with 50 μM Cl4-amidine, a PAD3 specific inhibitor. Following treatment, cells were lysed and equal concentrations of lysates examined by western blots following the AMC protocol. Our results show that inhibition of PAD3 results in a decrease in the level of citrullinated proteins in CID-9 cells (Fig 2). This is the first study to show that PAD3 catalyzes the citrullination of proteins in a mammary epithelial cell line.

#### Prolactin stimulation of CID-9 cells increases PAD3 expression

**Fig 3:**
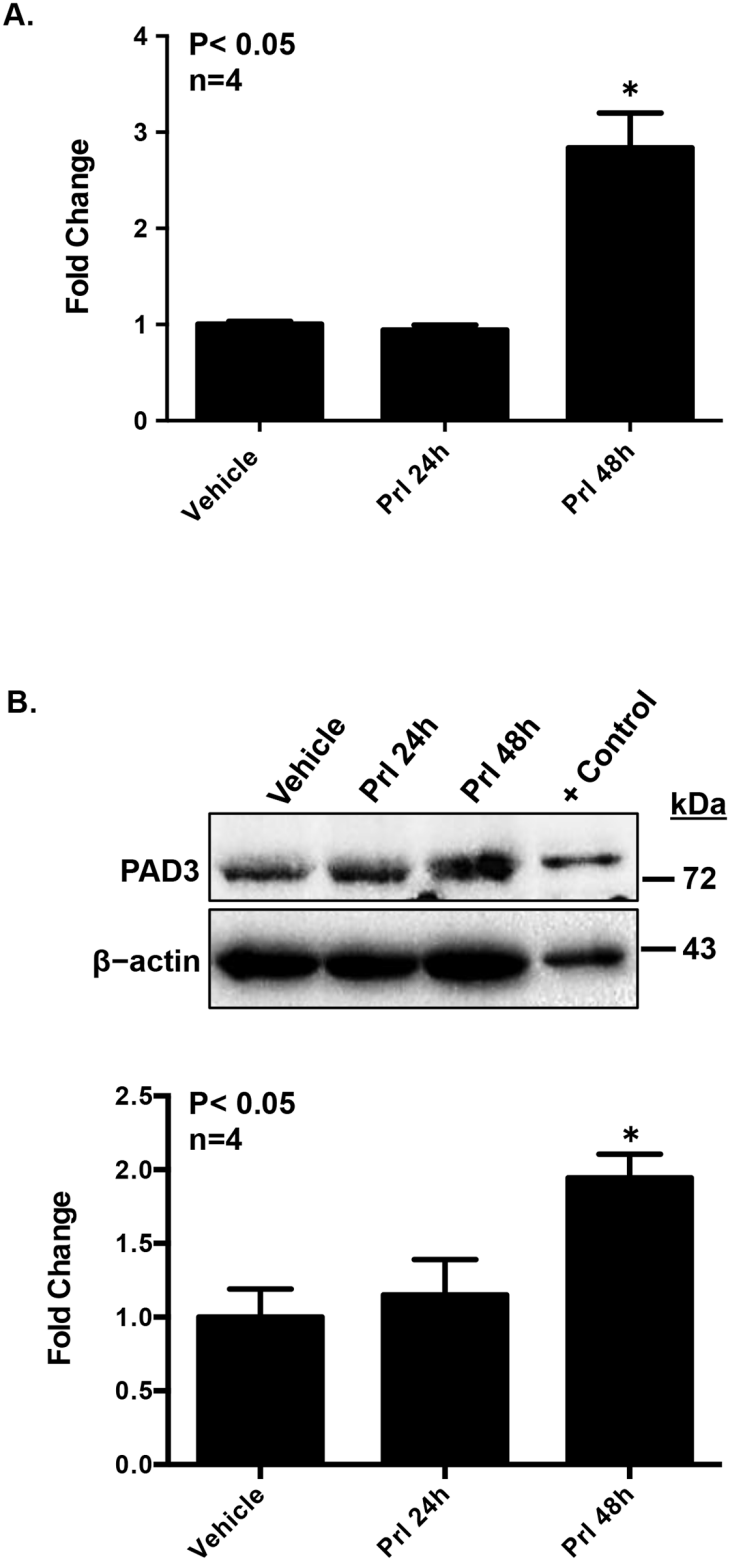
Prolactin stimulation of CID-9 cells increases PAD3 mRNA and protein expression. Equal numbers of CID-9 cells were grown in phenol red free media with charcoal-stripped FBS overnight. The following morning cells were treated with vehicle or 5 μg/mL of prolactin for 24 or 48 hours. (A) Prolactin treatment significantly increases PAD3 mRNA. Total RNA was extracted from CID-9 cells, reverse transcribed, and resulting cDNA examined by qPCR with intron spanning primers specific for PAD3 or GAPDH as the reference gene control. All data values were normalized to vehicle treated control and data are presented as means ± SEM. Means were separated using SNK ANOVA and * indicates significant difference (P<0.05). (B) Prolactin treatment increases PAD3 protein levels. The top panel shows a representative western blot, while the graph in the bottom panel represents the quantification of western blots using BioRad Image Lab 4.0 (n = 4, P<0.05). Protein concentrations of cell lysates were determined by BCA assay and equal amounts loaded and examined by western blot. Membranes were probed with a rabbit anti-PAD3 antibody or with anti-β-actin as a loading control. The positive control was generated by overexpressing human PAD3 in CID-9 cells.

Since prolactin is an important hormone in mammary gland physiology, we hypothesized that prolactin treatment of CID-9 cells would stimulate PAD3 expression. CID-9 cells were chosen for this experiment because they show a strong prolactin induced response in lactation related gene expression such as β-casein [17]. CID-9 cells were treated with vehicle or 5 μg/mL of prolactin for 24 or 48 hours, and PAD3 mRNA levels measured by qPCR. Prolactin stimulation of CID-9 cells for 48 hours significantly increased PAD3 mRNA by approximately 2 fold versus vehicle treated cells (P<0.05) (Fig 3A). Next, CID-9 cells were treated with 5 μg/mL of prolactin for 24 or 48 hours, lysed, and equal concentrations of lysates examined by western blots probed with an anti-PAD3 antibody (Fig 3B). The anti-PAD3 antibody used in both western blots and IHC is specific for PAD3 and does not appear to cross-react with other PAD family members (S2 Fig). Consistent with our qPCR results, quantification of blots revealed that 48 hours of prolactin treatment significantly increased PAD3 protein levels (P<0.05). Following the same treatment paradigm, PAD3 expression in CID-9 cells was also examined by IF confocal microscopy (S3 Fig). The representative images illustrate that prolactin stimulation of CID-9 cells for 48 hours increases PAD3 expression levels in the cytoplasmic and nuclear compartment (h) compared to vehicle treated controls (e). Taken together, our results show for the first time that prolactin can stimulate PAD3 expression.

#### Prolactin activates the JAK2/STAT5 signaling pathway to stimulate PAD3 expression

**Fig 4:**
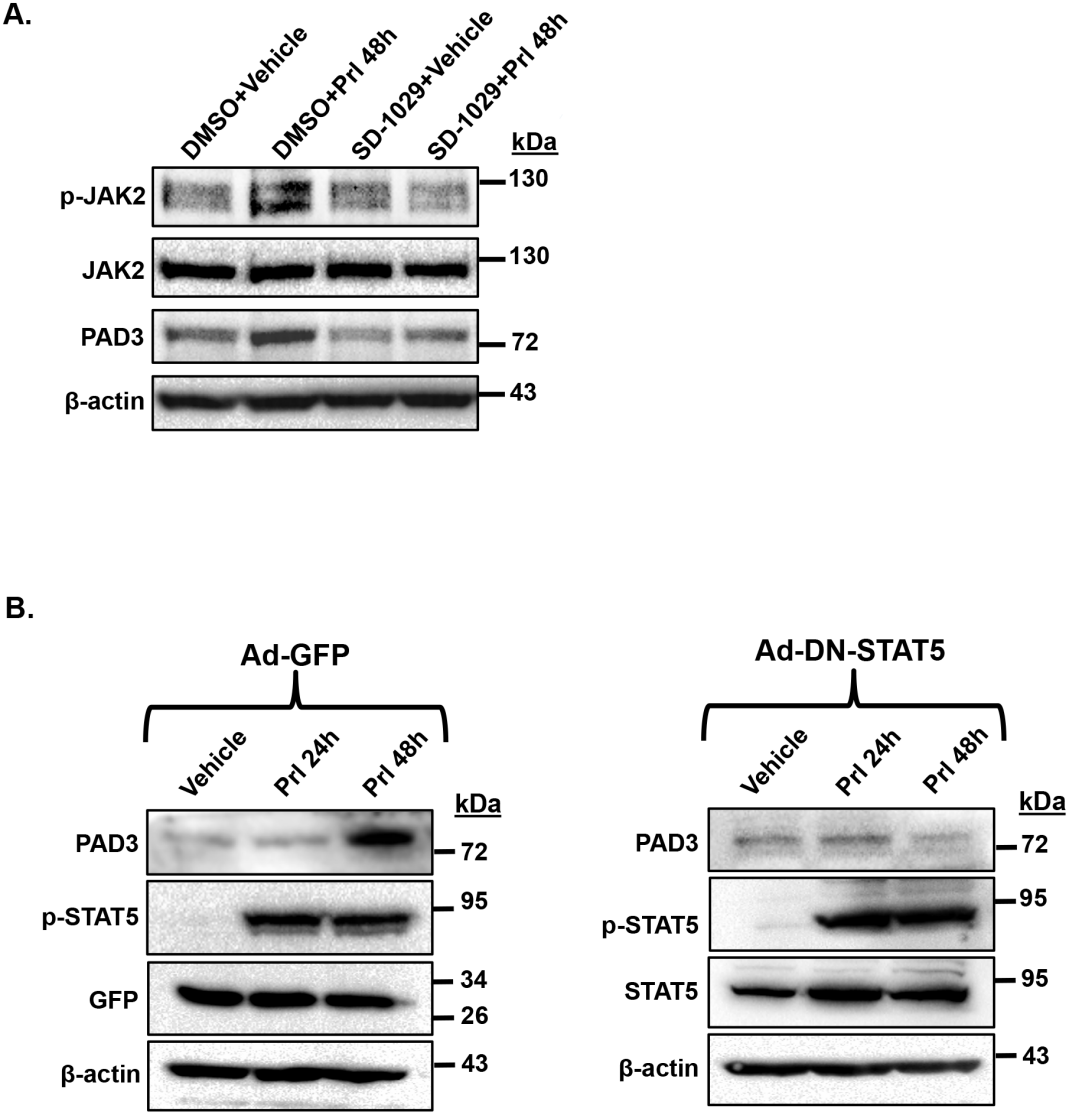
Prolactin stimulates the JAK2/STAT5 signaling pathway to upregulate PAD3 expression in CID-9 cells. (A) JAK2 mediates prolactin induced PAD3 upregulation. CID-9 cells were pre-treated for 4 hours with DMSO or 3 μM JAK2 inhibitor (SD-1029). Following pre-treatment, cells were stimulated with vehicle or 5 μg/mL of prolactin for 48 hours. After the first 24 hours, cells received fresh inhibitor. Protein concentrations of cell lysates were determined by BCA assay and equal amounts examined by western blot. Membranes were probed with anti-PAD3, p-JAK2, total JAK2 antibodies or with anti-β-actin as a loading control. (B) STAT5 mediates prolactin induced PAD3 upregulation. CID-9 cells were infected overnight with 10 MOI of an adenovirus expressing GFP (Ad-GFP) or an adenovirus expressing a dominant-negative form of STAT5 (Ad-DN-STAT5) as indicated. The following morning cells were treated with vehicle or 5 μg/mL of prolactin for 24 or 48 hours. Protein concentrations of cell lysates were determined by BCA assay and equal amounts examined by western blot. Membranes were probed with anti-PAD3, GFP, p-STAT5, total STAT5 or β-actin antibodies.

In mammary secretory cells, prolactin activation of the JAK2/STAT5 signaling pathway is critical for gene expression during lactation. To define the signaling mechanism through which prolactin induces PAD3 expression, CID-9 cells were pre-treated with DMSO or 3 μM JAK2 inhibitor SD-1029 for 4 hours, followed by treatment with vehicle or 5 μg/mL of prolactin for 48 hours. Inhibitor was re-applied at the same concentration after the first 24 hours. Equal concentrations of protein lysates were examined by western blots and membranes were probed with anti-phosphorylated JAK2 (p-JAK2), total JAK2, PAD3, and β-actin antibodies. To verify inhibition of JAK2 activity in our paradigm, vehicle and prolactin stimulated CID-9 cells were examined for phosphorylation of JAK2 on tyrosine residue 221 in the presence and absence of the JAK2 inhibitor. With DMSO, prolactin treatment for 48 hours increased p-JAK2, while inhibitor treatment attenuated p-JAK2 at 48 hours (Fig 4A). In the DMSO control, treatment of CID-9 cells with prolactin for 48 hours increases PAD3 expression. In contrast, treatment of CID-9 cells with the JAK2 inhibitor attenuates prolactin induced PAD3 expression at 48 hours (Fig 4A). These results suggest that JAK2 can mediate prolactin induced PAD3 expression.

We next focused on STAT5, which is a well-defined downstream target of JAK2 in mammary secretory cells. CID-9 cells were infected with adenovirus (MOI = 10) expressing a dominant-negative form of STAT5 (Ad-DN-STAT5) or GFP (Ad-GFP) as a control. The DN-STAT5 construct is a naturally occurring truncation of STAT5 at residue 713 which deletes the C-terminal transactivation domain resulting in equal loss of transcriptional activity of STAT5a and STAT5b [27]. Following an overnight infection, cells were treated with 5 μg/mL prolactin for 24 or 48 hours. In the Ad-GFP infected cells, 48 hours of prolactin stimulation resulted in an increase in PAD3 expression and a corresponding increase in p-STAT5 (Fig 4B). Following infection of CID-9 cells with Ad-DN-STAT5, prolactin stimulation still resulted in phosphorylation of STAT5 at tyrosine residue 694 at 24 and 48 hours; however, due to the deletion of the transactivation domain, prolactin induced PAD3 expression was blunted at 48 hours (Fig 4B). Collectively, these results indicate that prolactin induced PAD3 expression in CID-9 cells can be mediated by the JAK2/STAT5 signaling pathway.

#### The human *PAD3* gene promoter is prolactin responsive in CID-9 cells

**Fig 5:**
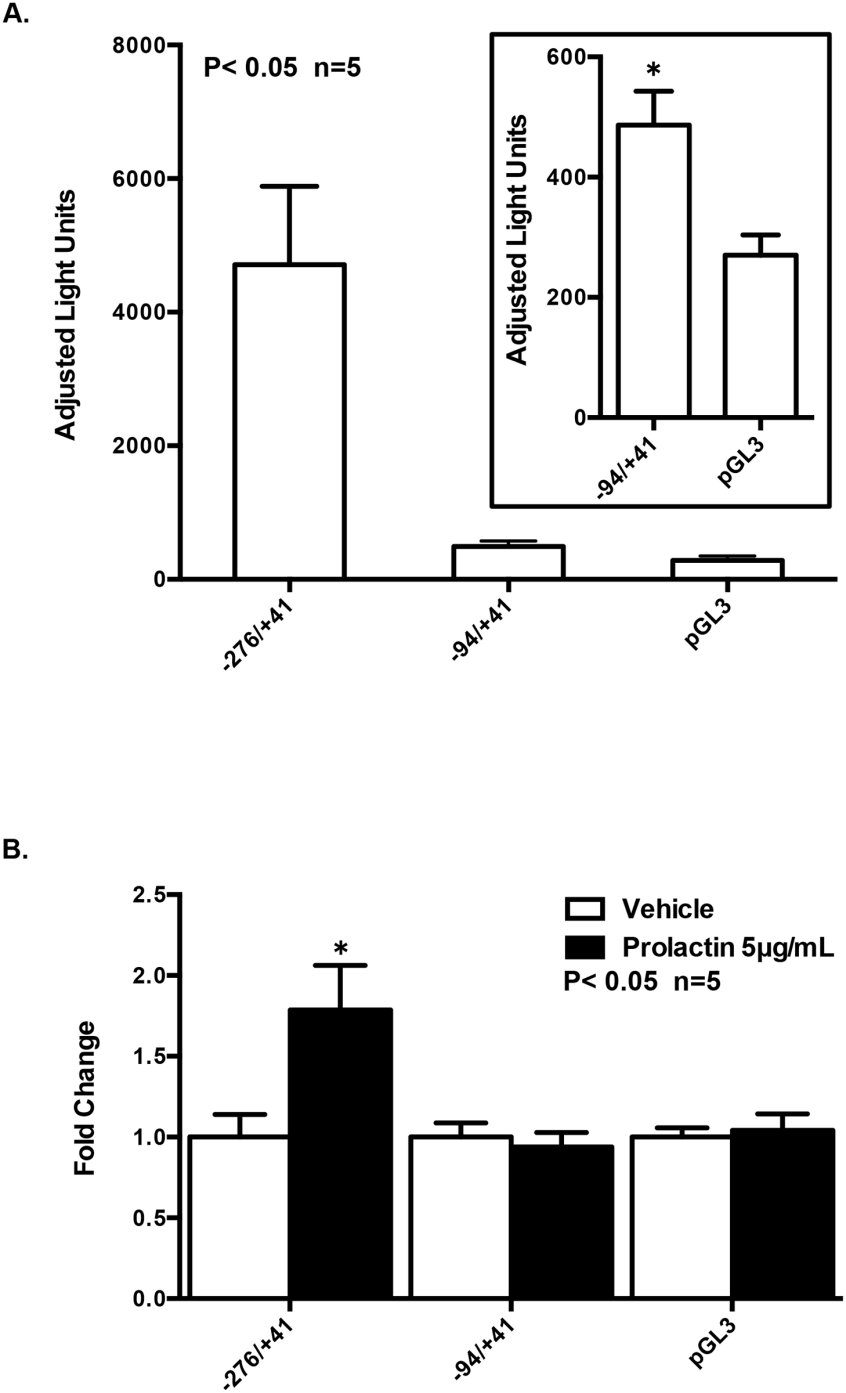
The human *PAD3* gene promoter is prolactin responsive in CID-9 cells -276/+41 and -94/+41 base pairs of the human *PAD3* gene promoter were cloned from genomic DNA and fused to the cDNA of luciferase. CID-9 cells were transfected overnight with hPAD3–276/+41-Luc, hPAD3–94/+41-Luc, pGL3 empty, and CMV-β-galactosidase. The next morning cells were treated with vehicle or 5 μg/mL of prolactin for 24 hours. Cellular lysates were assayed for luciferase and β-galactosidase activity. Luciferase values were corrected for β-galactosidase activity, and data are expressed as adjusted light units (top panel A) or fold change in adjusted luciferase activity (bottom panel B). The inset figure in top panel A illustrates that the hPAD3–94/+41-Luc construct has significant transcriptional activity compared to the pGL3 empty. Values represent the mean ± SEM. Means were separated using Student’s T-Test with * designating significant differences with treatment (P<0.05).

Upon activation by JAK2, phosphorylated STATs dimerize, translocate to the nucleus, and bind GAS motifs on lactation gene promoters. Given that JAK2 and STAT5 can mediate the prolactin induced increase in PAD3 expression, we next examined this response at the level of the human *PAD3* gene promoter. Human PAD3 (hPAD3) promoter constructs of -276/+41 and -94/+41 were fused to the cDNA of luciferase and verified by sequencing. PAD3 promoter-luciferase and CMV-β-galactosidase constructs were transiently transfected into CID-9 cells overnight and the following morning treated with vehicle or 5 μg/mL of prolactin. The hPAD3–276/+41 and -94/+41 luciferase constructs are transcriptionally active compared to pGL3 empty, although a significant decrease in promoter activity occurs with truncation to -94 bp (Fig 5A). Prolactin stimulation of CID-9 cells transfected with the hPAD3–276/+41 construct results in a significant increase in luciferase activity compared to vehicle treated control (P<0.05); however, this response is lost in the hPAD3–94/+41 construct (Fig 5B). Thus, the 182 bp region between -276 and -94 appears necessary for prolactin induced expression of the human *PAD3* gene promoter in mammary epithelial cells. Our results are the first to show prolactin responsiveness of a PAD gene promoter.

#### PAD3 expression in mouse mammary tissue initiates in late pregnancy and is highest during lactation

**Fig 6:**
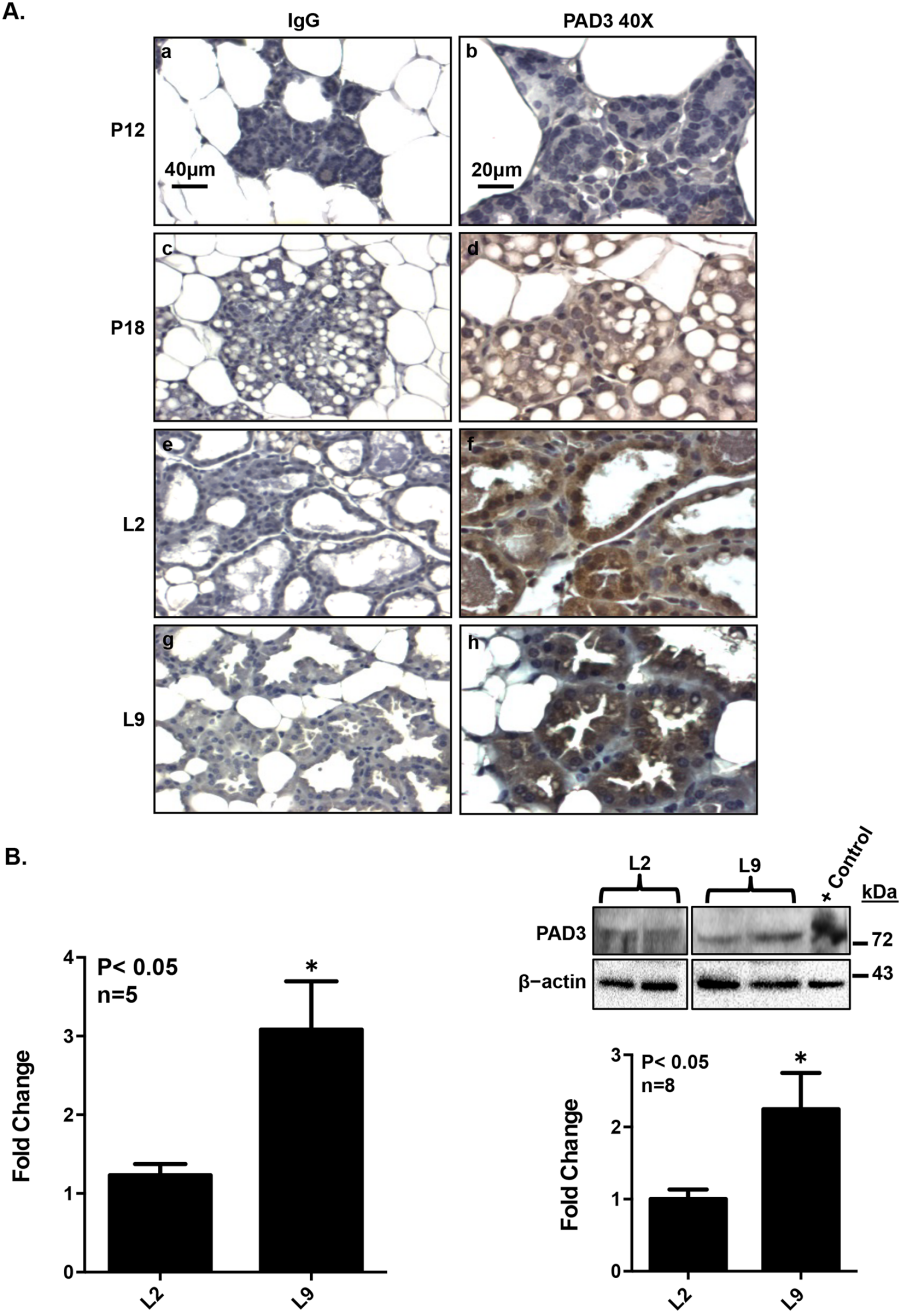
PAD3 expression in mouse mammary secretory cells initiates during late pregnancy and is highest during lactation. (A) PAD3 expression is first detected at P18 and increases from L2 through L9. Mouse mammary tissue from pregnancy day 12 (a, b), day 18 (c, d), lactation day 2 (e, f), and day 9 (g, h) was harvested, fixed in 10% neutral buffered formalin, embedded in paraffin and sectioned. 5 μm mammary tissue sections were subject to a standard IHC protocol using a rabbit anti-PAD3 antibody or an equal amount of non-specific rabbit IgG as a control. Images were taken with 20 and 40X objectives, and DAB staining represents PAD3 expression. (B) PAD3 mRNA and protein expression increases two-fold between L2 and L9. Total RNA was extracted from L2 and L9 mouse mammary glands, reverse transcribed, and resulting cDNA examined by qPCR with intron spanning primers specific for PAD3 or EE2F as the reference gene control. Data values were normalized to L2 to yield fold change, and data are expressed as means ± SEM. L2 and 9 mammary glands were homogenized in RIPA buffer and equal concentrations subject to western blot. The positive control was generated by overexpressing PAD3 in CID-9 cells. Western blots were quantified using BioRad Image Lab 4.0. Data values were normalized to L2 to yield fold change, and data are expressed as means ± SEM. Means were separated using Student’s T-test and * indicates a significant difference (P< 0.05).

Given that prolactin regulates PAD3 expression in CID-9 cells, we next examined if it is expressed in the lactating mouse mammary gland when serum prolactin levels are elevated. Published microarray data (GDS2843) generated from mouse mammary glands collected during pregnancy, lactation, and involution indicates that PAD3 mRNA increases towards the end of pregnancy and peaks on L9 [11]. To confirm this finding, mammary tissue was collected from mice at P12, P18, L2 and L9 and subject to a standard IHC protocol using an anti-PAD3 antibody or an equal mass of non-specific rabbit IgG. In the mouse mammary gland, PAD3 protein expression is first detected at P18 (d), increases at L2 (f) and peaks by L9 (h) (Fig 6A). Since PAD3 appears highest during lactation, we next quantified PAD3 mRNA and protein levels in lactating mammary glands. Both PAD3 mRNA and protein increase approximately 2 fold between L2 and L9 (P<0.05) (Fig 6B). Collectively, these results show that PAD3 expression initiates during late pregnancy and peaks on L9 in mouse mammary secretory cells.

#### Proteins in lactating mouse mammary glands are citrullinated, in part, by PAD3

**Fig 7:**
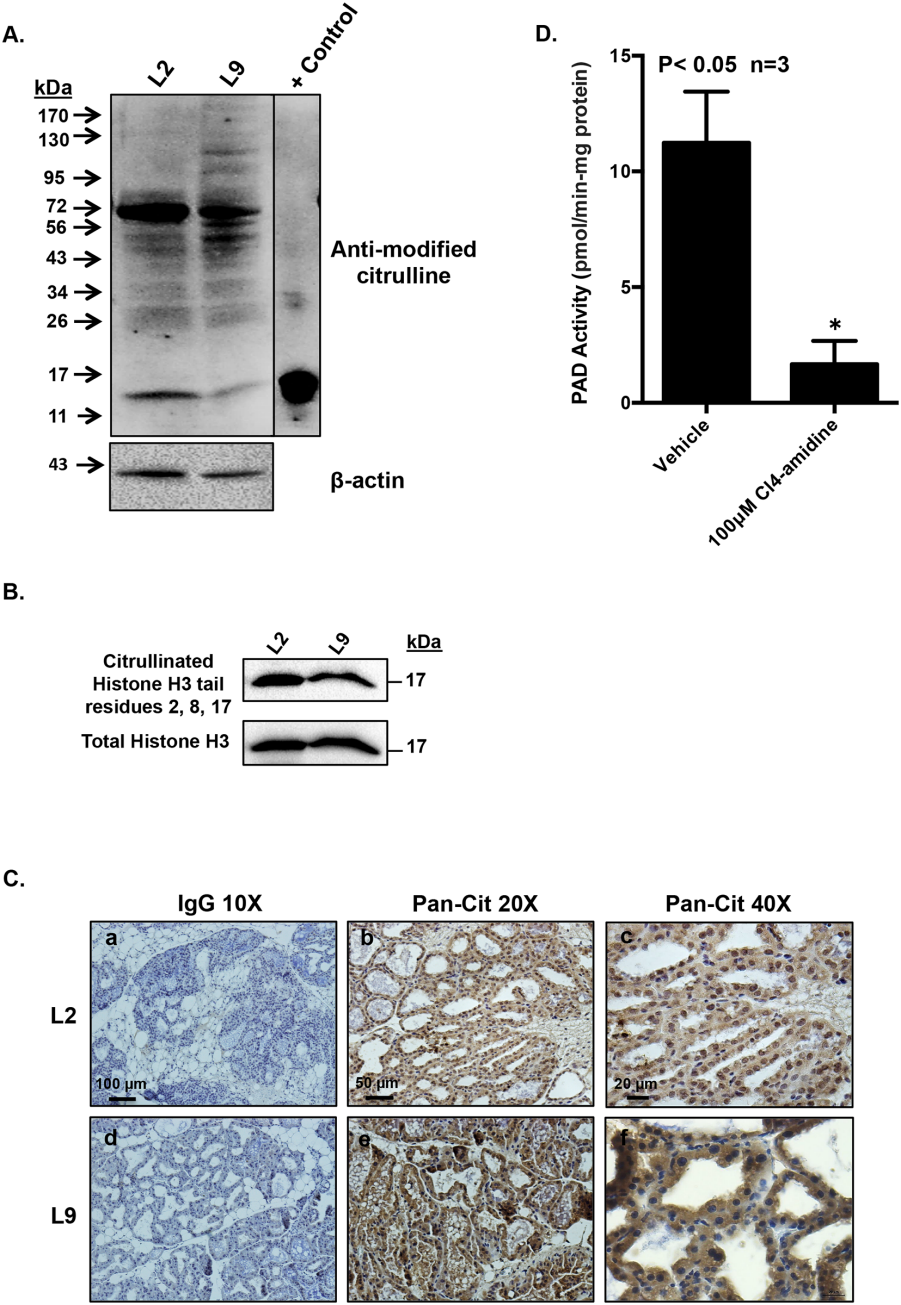
Citrullinated proteins are present in the lactating mammary gland and are citrullinated, in part, by PAD3. Mammary glands were collected on L2 and 9, homogenized and sonicated in modified RIPA buffer. (A) Citrullinated proteins are present in L2 and L9 mammary glands. Equal concentrations of protein lysates were examined by western blot following the AMC protocol. The positive control is *in vitro* citrullinated bulk histones. (B) Citrullinated histone tail arginine residues 2, 8, and 17 are present in L2 and L9 mammary glands. Equal concentrations of protein lysates were examined by western blot using an anti-citrullinated histone H3 arginine 2, 8, and 17 antibody. (C) Citrullinated proteins are detected in L2 and L9 mammary tissue. Mouse mammary tissue from lactation day 2 (a, b, c), and day 9 (d, e, f) was harvested, fixed in 10% neutral buffered formalin, embedded in paraffin and sectioned. 5 μm mammary tissue sections were subject to a standard IHC protocol using a rabbit anti-citrulline antibody or an equal amount of non-specific rabbit IgG as a control. Images were taken with 20 and 40X objectives, and DAB staining represents citrullinated proteins. (D) Cl4-amidine decreases PAD3 activity in L9 mammary gland lysates. Equal concentrations of L9 mammary gland lysates were incubated with 100 μM Cl4-amidine for 15 minutes then subject to COLDER PAD activity assay. Values represent the mean ± SEM. Means were separated using Student’s T-Test with * designating significant differences with treatment (P<0.05).

If timing of PAD3 expression is correct, then one would predict the presence of citrullinated proteins in the lactating mammary gland. To test this, mammary glands from L2 and L9 mice were excised, homogenized and sonicated in modified RIPA buffer. Equal concentrations of protein lysates were examined using the AMC western blot method. Citrullinated proteins were detected in both the L2 and L9 mammary glands (Fig 7A). The L2 and L9 lysates contain lower molecular weight bands that correlate in size with *in vitro* citrullinated bulk histones used as a positive control. To further investigate this finding, L2 and L9 lysates were examined by western blot and membranes probed with an antibody that detects citrullinated arginine residues 2, 8, and 17 on histone H3 tails. Citrullinated histones were detected in the L2 and L9 lysates (Fig 7B). L2 and L9 mammary glands were also examined by IHC with an anti-citrulline antibody. Citrullinated proteins were detected in mammary secretory cells in both the L2 and L9 mammary gland sections compared to IgG treated controls (Fig 7C). Next, we attempted to determine the relative contribution of PAD3 catalyzed citrullination in the L9 mammary glands. L9 mammary gland lysates were pre-incubated for 15 minutes with 100 μM Cl4-amidine, a PAD3 specific inhibitor, followed by the COLDER assay which measures PAD activity. PAD activity in the L9 samples was significantly reduced in the presence of Cl4-amidine (Fig 7D). Our results suggest that PAD3 can citrullinate proteins in the lactating mouse mammary gland.

## Discussion

The regulation of PAD expression and the function of protein citrullination in normal mammary gland physiology is not well understood. To increase our understanding of this, we first examined the expression of PAD isoforms 1–4 in mouse mammary epithelial CID-9 cells. In the cell line PAD3 expression is significantly elevated compared to other family members. Given this novel data, we chose to focus on PAD3, rather than other PADs, since PAD3 has never been characterized in mammary cell lines or tissue.

At the beginning of lactation, high volume milk production by mammary secretory cells, termed secretory activation, is initiated by decreasing serum progesterone concurrent with elevated prolactin. Previous work suggests that PAD expression and activity are high during pregnancy and lactation when serum prolactin levels are elevated. Therefore, we tested the hypothesis that prolactin stimulates PAD3 expression. Stimulation of CID-9 cells with prolactin increases PAD3 mRNA and protein expression approximately 2 fold. Our work also shows that PAD3 citrullinates proteins in CID-9 cells, although the identity of these citrullinated proteins is currently unknown. Interestingly, our IF studies indicated that PAD3 is expressed in the nucleus of CID-9 cells. This finding is not without precedent as PAD3 is localized to the nucleus in neurons from mixed rat cerebellar cultures and in chick spinal chords [22, 28, 29]. Currently, it is unclear how PAD3 translocates to the nucleus as is the identity of its catalytic targets in mammary epithelial cell nuclei.

We next investigated if prolactin activates the JAK2/STAT5 pathway to increase PAD3 expression. Treatment with the JAK2 inhibitor attenuates prolactin induction of PAD3 expression in CID-9 cells. Although attenuated, it does not eliminate PAD3 expression suggesting that other mechanisms are still capable of regulating basal levels of PAD3 expression. One such mechanism may be the liver X receptor (LXR)-retinoic acid receptor (RXR) signaling pathway which regulates PAD3 expression in keratinocytes [30]. In addition, SD-1029 is a JAK2 selective inhibitor, but may induce minimal off target effects on phosphorylation of JAK1 and SRC, so we cannot completely rule out their potential contribution to PAD3 expression. It is established that STAT5 deletion in mice results in lactation failure and that this transcription factor is critical for regulating expression of milk proteins such as β-casein in mammary secretory cells [31, 32]. In accordance with this, we found that STAT5 can mediate prolactin induced PAD3 expression in CID-9 cells. Given that the DN-STAT5 adenovirus lacks the C-terminal transactivation domain, our data suggests that this region is critical for transcriptional activation of the PAD3 gene. The STAT5 C-terminal transactivation domain can interact with p300/CREB binding protein to enhance prolactin mediate transcription [33]; therefore, a similar mechanism maybe required for PAD3 transcription. Lastly, it should be noted that we chose to focus on JAK2 and STAT5 because of their well-defined role in mediating prolactin induction of lactation related gene expression in mammary secretory cells; however, we cannot rule out the contributions of other JAK and STAT isoforms that may be present in the CID-9 cells.

To complement our analysis of the signaling pathway, we next investigated if prolactin stimulates activity of the human *PAD3* gene promoter. The human PAD3 promoter is transcriptionally active and prolactin responsive in the CID-9 cell line. Our results are the first to show that prolactin can regulate gene expression of a PAD family member. In our studies, the sequence located between -276 and -94 base pairs upstream of the transcriptional start site is necessary to mediate prolactin responsiveness of the human *PAD3* gene promoter in CID-9 cells. Previous work in human keratinocyte (NHEK) cells also found that truncation of the proximal human *PAD3* gene promoter to -94 significantly reduced basal activity [26]. Deletion from -276 to -94 eliminates the CCAAT box 1 and GC box 1 DNA binding motifs in the PAD3 promoter suggesting that these elements may mediate prolactin induced activity in CID-9 cells. Mutation analysis shows that these two elements are critical for basal activity of the hPAD3–276/+41 promoter in keratinocytes. Our analysis and that by Dong *et al*. indicates that this sequence does not contain any consensus GAS motifs which bind STAT5; however, prolactin responsiveness of gene promoters can occur through cooperative binding events [26]. For example, STAT5 and CCAAT/enhancer-binding protein beta (C/EBPβ) cooperatively bind on the β-casein gene promoter to integrate prolactin signaling in mammary epithelial cells in the presence of activated glucocorticoid receptor [34]. Additionally, prolactin stimulation of MCF-7 cells activates STAT5 and, in conjunction with ERα, Sp1 and C/EBP, increases prolactin receptor expression [35]. Although further investigation is needed, C/EBPβ and Sp1 transcription factor binding to the CCAAT box 1 and GC box 1 DNA binding motifs, respectively, may help mediate prolactin induction of PAD3 expression.

To confirm the physiological relevance of our findings in CID-9 cells, we next examined PAD3 in the mouse mammary gland. Since PAD isoforms can show differences between mRNA and protein levels, we analyzed pregnant and lactating mammary glands and confirmed that PAD3 protein expression initiates on P18 and is highest at L9. If PAD3 is expressed in mouse mammary secretory cells during lactation, then one would predict the presence of citrullinated proteins as well. As predicted, citrullinated proteins are detected in the L2 and L9 mammary glands by AMC blots. Although the AMC method cannot distinguish between citrullinated versus carbamylated proteins, the blots suggest that specific proteins may be modified during different stages of lactation [36]. The detection of citrullinated proteins in the mammary gland blots was further supported by our IHC studies using a pan-citrulline antibody which also detects citrullinated proteins in L2 and L9 mammary secretory cells. Despite not identifying a specific PAD3 citrullinated protein, we nevertheless attempted to address the relative contribution of PAD3 to the citrullination of proteins in the L9 mammary glands using Cl4-amidine. PAD3 does significantly contribute to total PAD activity in the L9 sample. Although we cannot completely rule out that Cl4-amidine inhibits other PADs to a lesser extent, Cl4-amidine does have greater than 20 and 50 fold selectivity for PAD3 over PAD1 and PAD4, respectively [25]. Proteomic studies are currently underway to identify the full cohort of citrullinated proteins in the lactating mouse mammary gland, but previous work suggests potential target proteins for PAD3 catalyzed citrullination. In neuronal stem cells PAD3 associates with the intermediate filament vimentin and may regulate cytoskeletal organization through modification of arginine residues in the vimentin amino-terminal head domain [22, 37]. In keratinocytes, PAD3 citrullinates cytokeratins 1 and 10 [18]. Our studies show that histone H3 arginine residues 2, 8, and 17 are citrullinated in L2 and L9 mammary gland lysates. Therefore, it will be interesting to determine if expression of specific lactation-related genes is mediated by PAD catalyzed histone citrullination.

To our knowledge, these are the first studies that demonstrate PAD3 expression and the presence of citrullinated proteins in the mammary gland during lactation. Our studies also show that prolactin activation of the JAK2/STAT5 signaling pathway can regulate expression of a PAD isoform. To date, no studies have examined the expression or function of PAD3 in breast cancer cell lines or primary tumors. Our novel finding of PAD3 in the mammary gland suggests that investigation of PAD3 in breast cancer is warranted. In addition, estrogen, EGF and now prolactin all regulate PAD expression in mammary tissue and cell line models. All three ligands clearly have very important functional roles in breast cancer tumorigenesis [38, 39]. Overall, our work suggests that PAD catalyzed citrullination maybe an important post translational modification for normal mammary gland function.

## Supporting Information

S1 Fig PAD3 is the most highly expressed PAD isoform in mouse mammary epithelial COMMA-1D cells. PAD3 mRNA is highest in COMMA-1D cells. Total RNA was extracted from COMMA-1D cells, reverse transcribed, and resulting cDNA examined by qPCR with intron spanning primers specific for PAD1, PAD2, PAD3, PAD4 or GAPDH as the reference gene control. All data values were normalized to PAD1 to yield fold change, and data are expressed as means ± SEM. Means were separated using Tukey’s test ANOVA and letters indicate significant differences (P< 0.05).
(TIF)

S2 Fig The PAD3 antibody is not cross reactive with other PAD isoforms. The anti-PAD3 antibody detects overexpressed human PAD3 and endogenous mouse PAD3 at the correct molecular weight in CID-9 cells. Mammalian expression plasmids containing the cDNAs for human PADs 1, 2, 3, and 4 were transfected into CID-9 cells. The following day, cellular lysates were harvested and equal concentrations were analyzed by western blot using an anti-PAD3 antibody. Membranes were stripped and re-probed with an anti-β-actin antibody to ensure equal loading.
(TIF)

S3 Fig Prolactin stimulates PAD3 expression in CID-9 cells compared to vehicle treated controls. CID-9 cells were fixed, permeabilized and subjected to IF using anti-rabbit PAD3 antibody (green) or an equal mass of a non-specific rabbit IgG. Cells were then stained with DAPI (Blue) and imaged at 40X with a confocal microscope.
(TIF)
