# Supplementary figures and images for: Peptidylarginine Deiminase 3 (PAD3) Is Upregulated by Prolactin Stimulation of CID-9 Cells and Expressed in the Lactating Mouse Mammary Gland
Source: PLoS One. 2016 Jan 22;11(1):e0147503. doi: 10.1371/journal.pone.0147503 (PMC4723263; doi:10.1371/journal.pone.0147503)

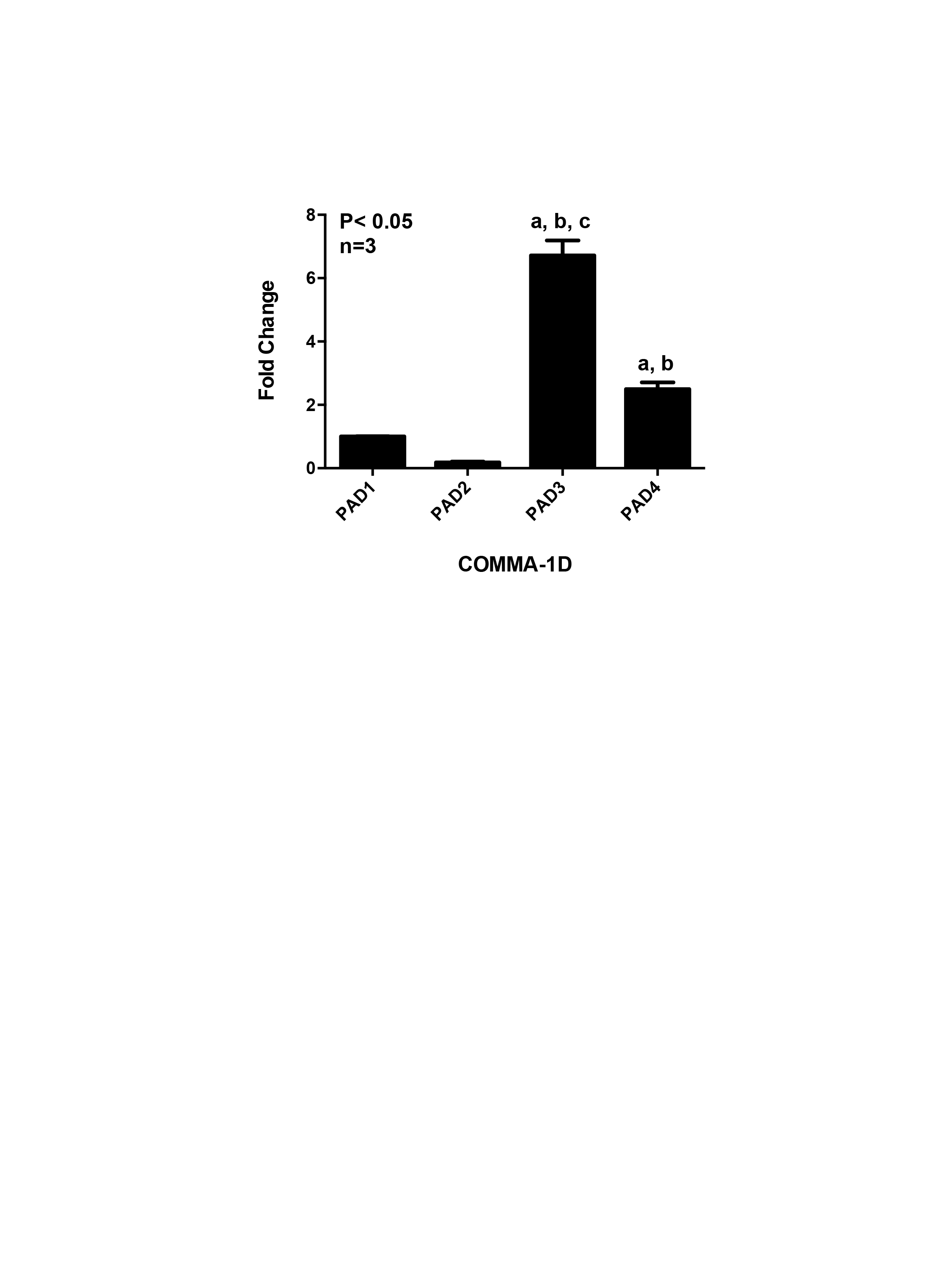

Supplement: S1 Fig — PAD3 mRNA is highest in COMMA-1D cells. Total RNA was extracted from COMMA-1D cells, reverse transcribed, and resulting cDNA examined by qPCR with intron spanning primers specific for PAD1, PAD2, PAD3, PAD4 or GAPDH as the reference gene control. All data values were normalized to PAD1 to yield fold change, and data are expressed as means ± SEM. Means were separated using Tukey’s test ANOVA and letters indicate significant differences (P< 0.05). (TIF) [file pone.0147503.s001.tif]

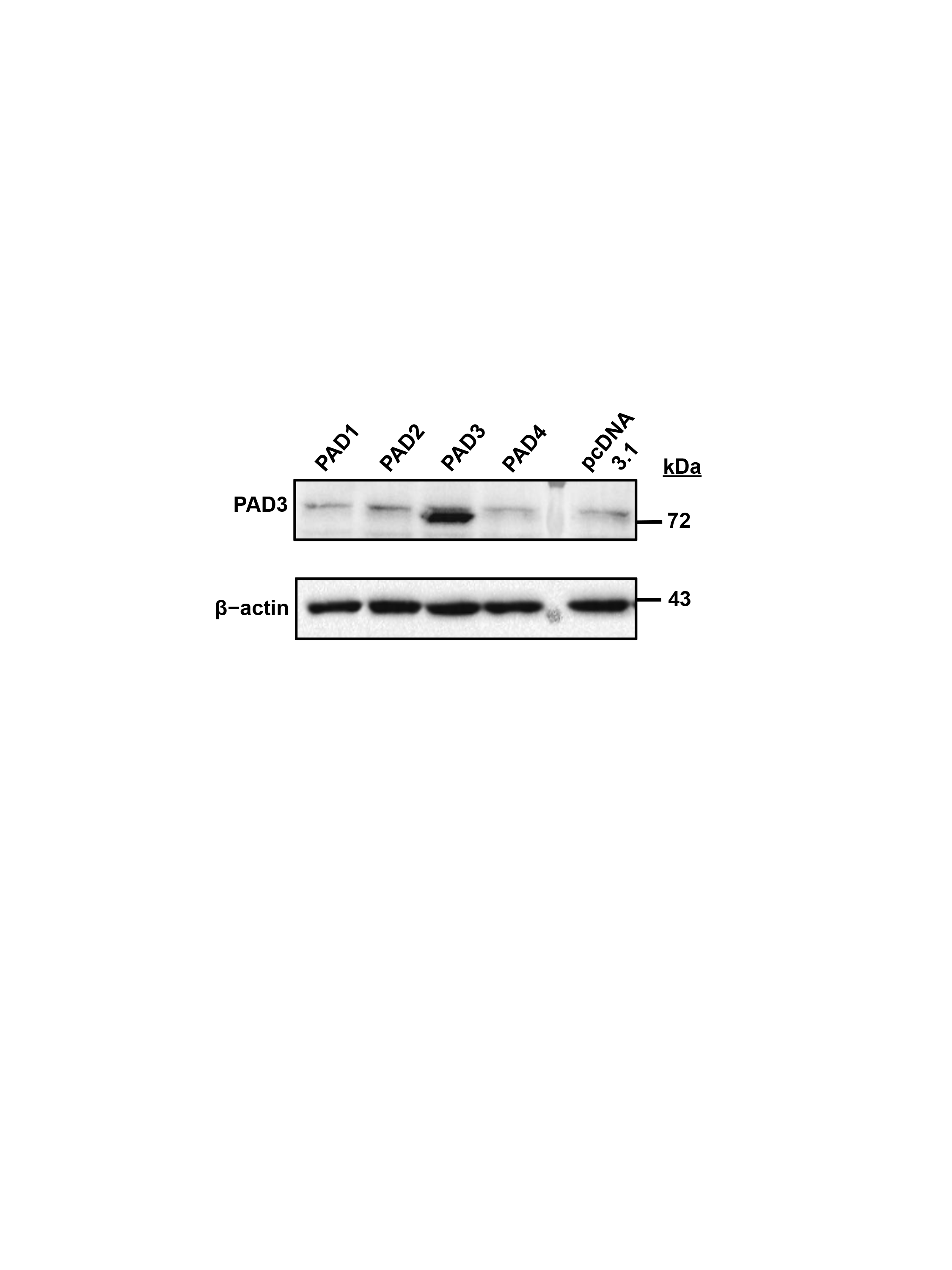

Supplement: S2 Fig — The anti-PAD3 antibody detects overexpressed human PAD3 and endogenous mouse PAD3 at the correct molecular weight in CID-9 cells. Mammalian expression plasmids containing the cDNAs for human PADs 1, 2, 3, and 4 were transfected into CID-9 cells. The following day, cellular lysates were harvested and equal concentrations were analyzed by western blot using an anti-PAD3 antibody. Membranes were stripped and re-probed with an anti-β-actin antibody to ensure equal loading. (TIF) [file pone.0147503.s002.tif]

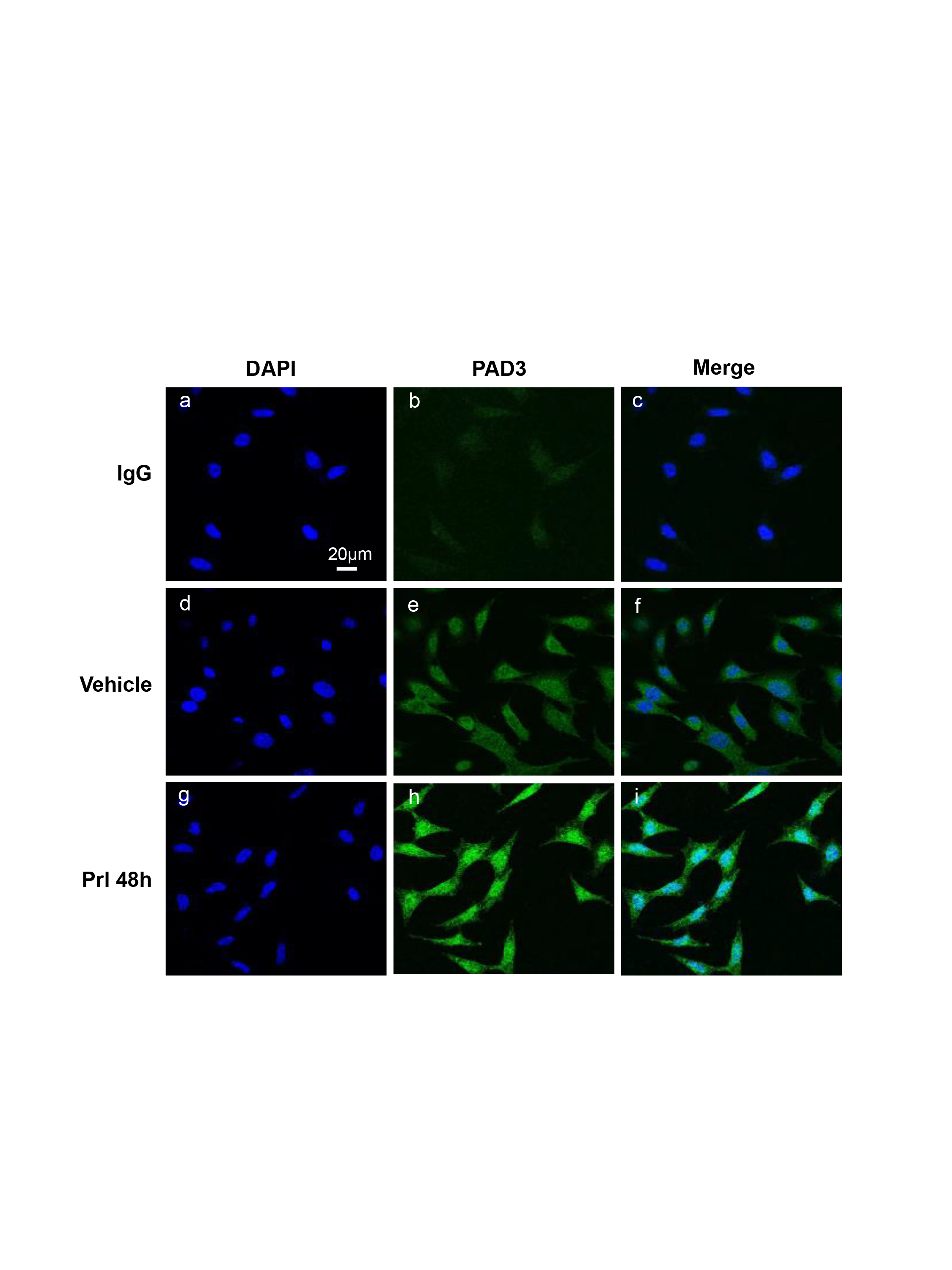

Supplement: S3 Fig — CID-9 cells were fixed, permeabilized and subjected to IF using anti-rabbit PAD3 antibody (green) or an equal mass of a non-specific rabbit IgG. Cells were then stained with DAPI (Blue) and imaged at 40X with a confocal microscope. (TIF) [file pone.0147503.s003.tif]
